# Combining MRI dual-sequence radiomics and clinical parameters predicts post-hypertriglyceridemic acute pancreatitis diabetes mellitus

**DOI:** 10.3389/fendo.2026.1887050

**Published:** 2026-08-03

**Authors:** Yanting Li, Xiyao Wan, Yuan Wang, Hangyu Li, Cui Tang, Wang Zeng, Xiaohua Huang

**Affiliations:** Department of Radiology, Affiliated Hospital of North Sichuan Medical College, Nanchong, China

**Keywords:** acute pancreatitis, hypertriglyceridemia, magnetic resonance imaging, pancreatogenic diabetes, radiomics

## Abstract

**Background:**

Post-acute pancreatitis diabetes mellitus (PPDM-A) represents the most prevalent subtype of diabetes secondary to exocrine pancreatic dysfunction. Patients with acute pancreatitis (AP) caused by hypertriglyceridemia (HTG-AP) face a high risk of PPDM-A. Since clinical predictors alone lack accuracy, integrating magnetic resonance imaging (MRI)-based radiomics may improve prognostic risk stratification.

**Methods:**

A retrospective cohort of 210 patients with HTG-AP was included and randomized into training and internal testing cohorts (n = 147 and 63, respectively; ratio 7:3). An independent external validation cohort (n = 119) from a separate hospital campus was also analyzed. Radiomics features from T2-weighted and late arterial phase contrast-enhanced T1-weighted MRI were selected via least absolute shrinkage and selection operator (LASSO) to generate a radiomics score (Rad-score). A random forest model that incorporated the Rad-score and independently significant clinical predictors was established. Model predictive ability was examined using receiver operating characteristic (ROC) curves, decision curve analysis (DCA), and reclassification metrics, including integrated discrimination improvement (IDI) and net reclassification improvement (NRI). SHapley Additive exPlanations (SHAP) analysis was employed to interpret feature contributions.

**Results:**

Seven optimal radiomics features were used to generate the Rad-score. The final combined model incorporated this score with three key clinical variables and achieved areas under the ROC curve (AUCs) of 0.905, 0.904, and 0.900 in the training, testing, and external validation cohorts, respectively, significantly outperforming single-modality models. SHAP analysis identified the Rad-score, length of hospital stay, high-sensitivity C-reactive protein, and recurrence of AP as principal predictive contributors.

**Conclusion:**

Integrating dual-sequence MRI radiomics with clinical features accurately predicts HTG-PPDM-A risk. Enhanced by SHAP interpretability, this non-invasive tool enables transparent long-term risk prediction to guide personalized interventions.

## 1 Introduction

Acute pancreatitis (AP), an inflammation process of the pancreas, frequently disrupts endocrine function (1). Epidemiological evidence suggests that approximately 20% of patients with AP subsequently develop post-AP diabetes mellitus (PPDM-A) (2, 3). In comparison with type 2 diabetes mellitus (T2DM), PPDM-A is linked with diminished glycemic control and elevated risk of pancreatic ductal adenocarcinoma (4–6). In clinical practice, PPDM-A is often misdiagnosed as T2DM, which may delay appropriate management strategies (5). Prognostic assessment of elevated risk is therefore vital for timely treatment and personalized care.

The risk of PPDM-A is strongly influenced by the underlying cause of AP (3). In particular, there has been a growing focus on hypertriglyceridemia-associated acute pancreatitis (HTG-AP) owing to its rising incidence and distinct pathophysiological features (7). In contrast to biliary or alcohol-related AP, HTG-AP involves excessive chylomicron accumulation and rapid elevation of free fatty acids (FFAs), which trigger oxidative stress and progressive pancreatic islet cell injury (8, 9). Consequently, patients with HTG-AP are at significantly greater risk of developing PPDM-A compared with other etiological subtypes (10). However, conventional laboratory markers mainly reflect systemic inflammation and are insufficient for detecting subtle, diffuse pancreatic damage (11), highlighting the need for more sensitive diagnostic approaches.

Multiparametric magnetic resonance imaging (MRI) affords a series of distinctive advantages in this prognostic setting. T2-weighted imaging (T2WI) offers sensitivity superior to that of computed tomography (CT) when detecting early interstitial edema and inflammatory exudation (12), while late arterial phase contrast-enhanced T1-weighted imaging (CE-T1WI) can capture microvascular perfusion abnormalities within pancreatic parenchymal in a dynamic fashion (13). Despite these benefits, routine MRI interpretation is largely qualitative and fails to fully leverage the rich quantitative information embedded in imaging data (14).

Radiomics addresses these imaging-related limitations through its ability to derive specific features from imaging scans, thereby allowing the identification of subtle spatial heterogeneity that is not visible to the naked eye (15). Although prior studies (16, 17) have explored radiomics-based prediction of PPDM-A, most have included heterogeneous patient populations, limiting their relevance to HTG-AP-specific mechanisms. In addition, these studies often lacked multimodal integration and external validation. Moreover, conventional machine learning approaches are often criticized for their limited interpretability, which restricts clinical adoption (18).

Accordingly, the present study was developed with the goal of designing and externally validating a predictive model for HTG-PPDM-A by integrating dual-sequence MRI radiomics with clinical data. Interpretability was enhanced by application of the SHapley Additive exPlanations (SHAP) framework to elucidate the contribution of individual features, thereby offering a transparent and clinically applicable tool for prognostic assessment of risk and personalized outpatient management.

## 2 Materials and methods

### 2.1 Participants

This retrospective, dual-center investigation enrolled individuals with HTG-AP treated at the Affiliated Hospital of North Sichuan Medical College between February 2022 and December 2024, forming the derivation cohort. An independent validation cohort was established using data from a geographically distinct campus (Women’s and Children’s Hospital Affiliated to North Sichuan Medical College) collected between January 2019 and June 2021. The study was approved by the Institutional Review Board (Approval No.: 2024ER94–1), with a waiver of informed consent as the study was retrospective. This study was designed, conducted, and reported in strict accordance with the Strengthening the Reporting of Observational Studies in Epidemiology (STROBE) statement for cohort studies (19). All analyses were conducted under blinded conditions.

Inclusion criteria were based on whether patients met the established diagnostic criteria for acute pancreatitis (AP), defined as the presence of at least two of the following three features (20): acute onset of persistent epigastric pain, serum lipase or amylase concentrations at least three times the upper limit of normal, or characteristic imaging findings of AP. Additionally, the etiology had to be attributed to HTG, defined as elevated serum triglyceride (TG) levels > 5.65 mmol/L (500 mg/dL) in the absence of other common etiologies such as gallstones or alcohol use (21, 22). Eligible patients also underwent upper abdominal MRI within seven days of hospital admission. Diabetes status was determined according to the American Diabetes Association (ADA) diagnostic criteria (6, 23), defined as any of the following: random plasma glucose ≥ 11.1 mmol/L, fasting plasma glucose ≥ 7.0 mmol/L, 2−hour plasma glucose ≥ 11.1 mmol/L during oral glucose tolerance testing, or HbA1c ≥ 6.5%, with confirmation required in individuals without overt symptoms.

PPDM−A was defined as new−onset diabetes meeting ADA criteria that developed three months after the onset of AP in patients without a prior history of diabetes (24). This 3−month interval was selected to avoid the confounding effects of acute−phase stress hyperglycemia. The occurrence of PPDM−A was ascertained through systematic review of electronic medical records and follow−up interviews conducted at three−month intervals (median follow−up: 30 months for the derivation cohort and 55 months for the external validation cohort).

**Figure 1:**
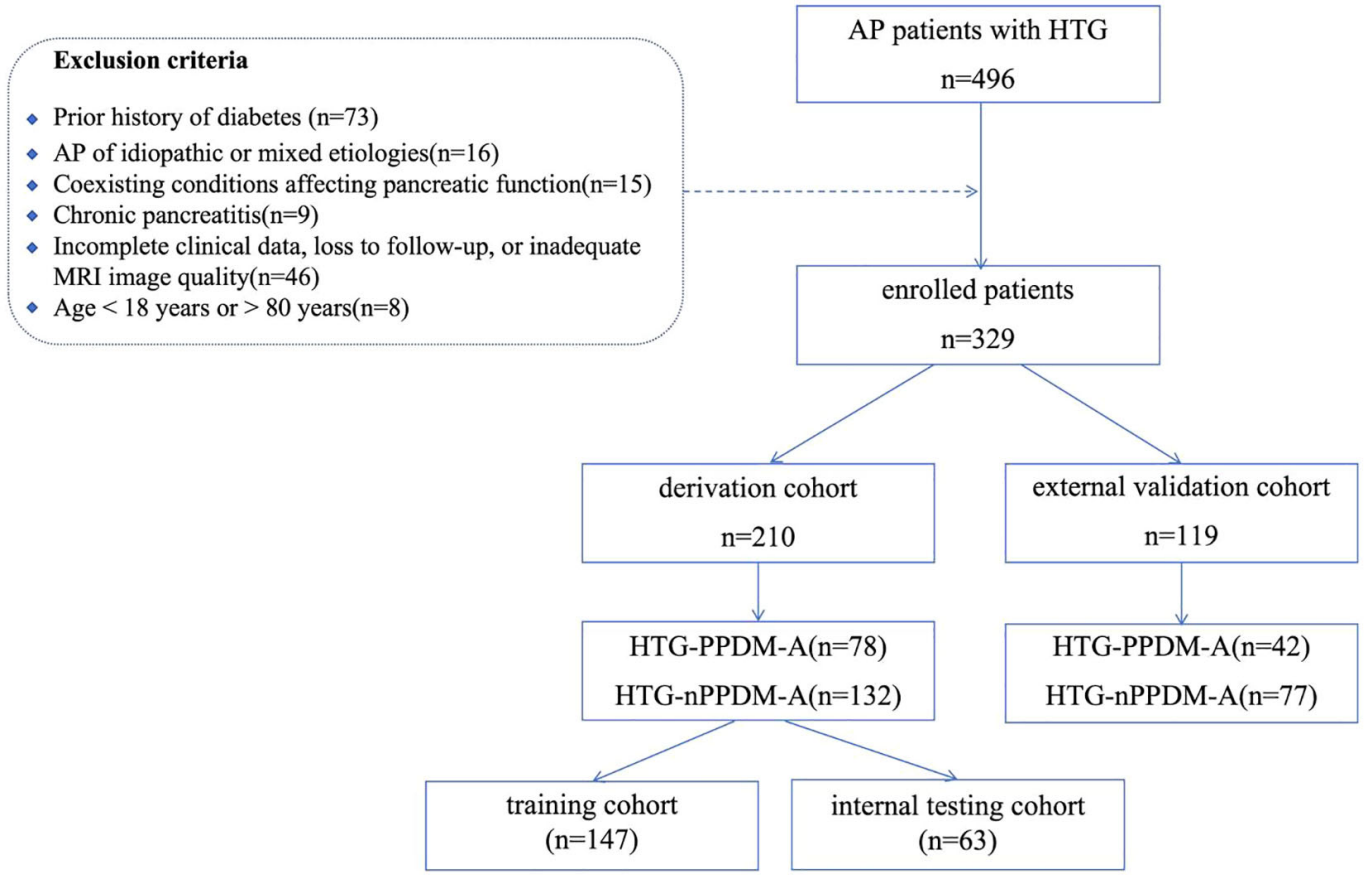
Flowchart of the patient selection process.

Patients were excluded if they had a prior diagnosis of diabetes mellitus, AP of mixed or unclear etiology, or coexisting conditions known to impair pancreatic function, such as previous pancreatic surgery, pancreatic tumors/trauma, or hemochromatosis (3). Additional exclusion criteria included chronic pancreatitis, incomplete clinical data, loss to follow-up, or poor-quality MRI images unsuitable for analysis. Individuals aged below 18 or above 80 were also not included. Patient selection is presented in **Figure 1**.

### 2.2 Clinical data

Clinical and laboratory variables were retrospectively obtained from electronic medical records for all enrolled patients. Three different categories of data were analyzed: (1) baseline characteristics, including sex, age, body mass index(BMI), alcohol consumption or smoking, presence of fatty liver disease, hypertension, prior biliary stones, and recurrence of AP; (2) clinical manifestations and outcomes, such as peritonitis, length of hospital stay, systemic inflammatory response syndrome (SIRS), ascites, and pleural effusion; and (3) admission laboratory indices, including white blood cell count, neutrophil percentage, serum calcium, pancreatic amylase, lipase, high-sensitivity C-reactive protein (hs-CRP), TG, total cholesterol, fasting blood glucose, and low-/high-density lipoprotein cholesterol. The extracted data were confirmed by two independent investigators.

### 2.3 MRI scanning

In the derivation cohort, both unenhanced and contrast-enhanced pancreatic MRI examinations were performed using a 16-channel phased-array body coil system (uMR790, United Imaging Healthcare, Shanghai, China). Standardized pre-scan preparation, including fasting and breathing training, was implemented for all patients. For the external validation cohort, imaging was conducted using a 32-channel body coil system (Discovery 750, GE Healthcare, USA). At both centers, T2WI and late arterial phase CE-T1WI were acquired in consistent anatomical planes. Details of the acquisition parameters are included in **Supplementary Table 1**. To ensure anatomical correspondence across sequences, CE-T1WI images were aligned via spatial registration.

### 2.4 Image segmentation and feature extraction

Independent radiomic analysis of both T2WI and CE-T1WI sequences was undertaken using 3D Slicer (v5.2.2). A radiologist who had over eight years’ experience in abdominal imaging manually delineated regions of interest (ROIs) along the pancreatic contours in a slice-by-slice manner (**Supplementary Figure 1**). The segmented regions included pancreatic parenchyma while explicitly excluding necrotic tissue, major vascular structures, and surrounding irrelevant tissues. To standardize image data, all scans were resampled to 1 × 1 × 1 mm3 isotropic voxels. Multi-scale texture features were extracted using Laplacian of Gaussian (LoG) filters with σ values of 0.5, 1.0, 1.5, and 2.0 mm. Intensity discretization was performed using a fixed bin width of 25 to reduce noise and enhance comparability, following the standardized image preprocessing guidelines (6, 25).

Feature extraction was conducted using the PyRadiomics platform, yielding 1,223 quantitative features per sequence. These included morphological descriptors, first-order statistics, and higher-order texture features from matrices such as the gray-level co-occurrence, gray-level run length, gray-level dependence, gray-level size zone, and neighborhood gray tone difference matrices. To evaluate reproducibility, a second radiologist independently re-segmented ROIs in 70 randomly selected cases after a two-week interval, blinded to clinical information. Features exhibiting an intraclass correlation coefficient (ICC) > 0.75 were kept for further analysis (26).

**Figure 2:**
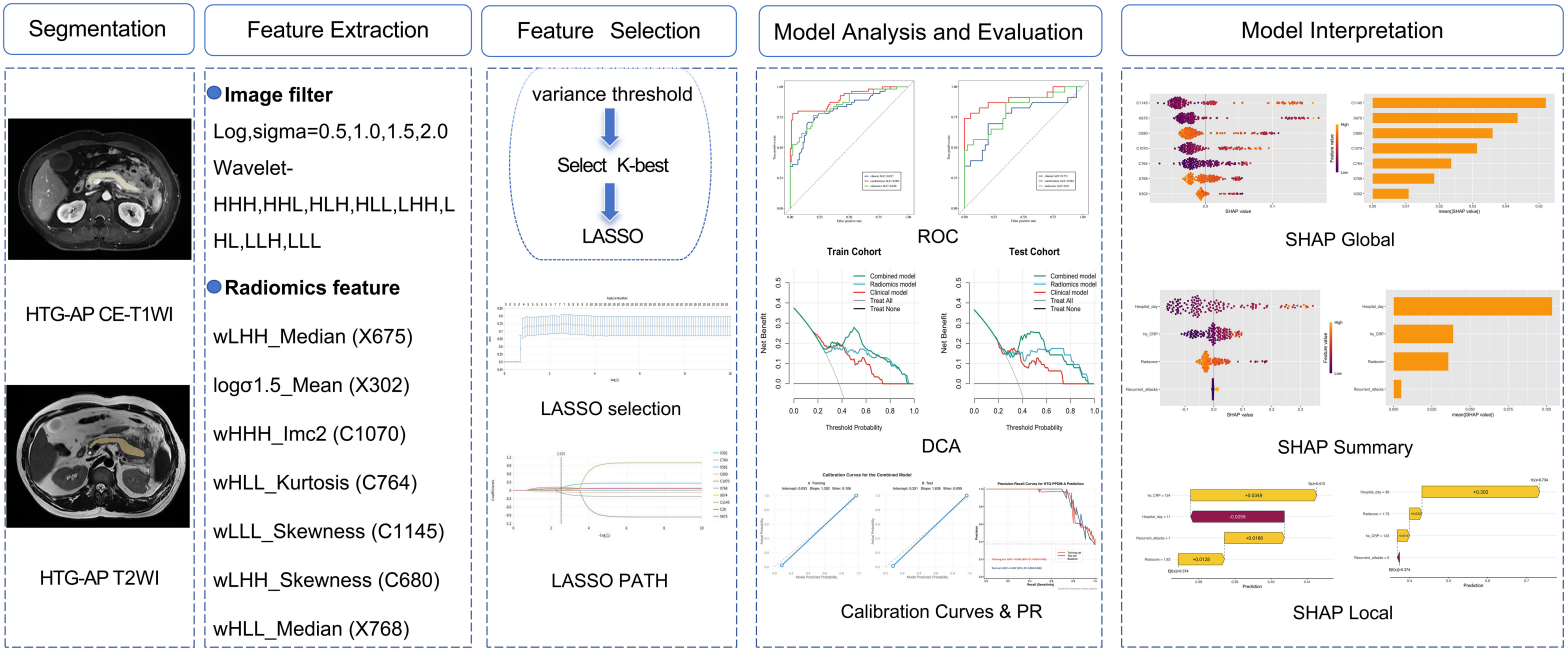
Radiomics workflow for the development, evaluation, and interpretation of the predictive model. The analytical pipeline consists of five main stages: (1) Segmentation: Three-dimensional regions of interest (ROIs) were manually delineated on dual-sequence MR images (CE-T1WI and T2WI). (2) Feature Extraction: High-throughput radiomic features were extracted from original and transformed images (Log and Wavelet filters). (3) Feature Selection: Optimal predictive features were identified through a stepwise dimensionality reduction process comprising variance thresholding, SelectKBest, and LASSO regression. The optimal LASSO regularization parameter (λ) was determined via 10-fold cross-validation. (4) Model Analysis and Evaluation: The predictive performance of the combined model was comprehensively assessed using ROC curves, PR curves, and calibration curves with Brier scores, while clinical utility was evaluated via DCA. (5) Model Interpretation: The internal decision-making logic of the models was elucidated using the SHAP framework, encompassing global feature importance, summary beeswarm plots, and local individual waterfall plots. HTG-AP, hypertriglyceridemia-associated acute pancreatitis; CE-T1WI, contrast-enhanced T1-weighted imaging; T2WI, T2-weighted imaging; Log, Laplacian of Gaussian; LASSO, least absolute shrinkage and selection operator; ROC, receiver operating characteristic; PR, precision-recall; DCA, decision curve analysis; SHAP, SHapley Additive exPlanations.

Feature reduction proceeded through multiple stages: variance filtering, SelectKBest selection, and the least absolute shrinkage and selection operator (LASSO) regression. The LASSO optimal regularization parameter (λ) was obtained using ten-fold cross-validation. Selected features were subsequently normalized using Z-score transformation. The complete analytical process is illustrated in **Figure 2**.

### 2.5 Model construction and evaluation

Patients from the derivation cohort were randomly assigned to training and testing cohorts (ratio 7:3) using stratified random sampling to preserve class distribution. Features were selected in the training cohort, followed by construction of the model, while the testing and external validation cohorts were used to assess model generalizability.

Three random forest (RF) models were developed. These were: (1) the clinical model, which incorporated the independently predictive factors determined from binary logistic regression; (2) the radiomics model, which incorporated only selected imaging features; and (3) the combined model, which included both clinical and radiomic variables. Model hyperparameters were predefined as follows: 100 decision trees, maximum tree depth of 2, and Gini impurity as the splitting criterion. To benchmark the performance of our RF-based model against other commonly used classifiers, we additionally trained support vector machine (SVM), logistic regression (LR), and Gaussian process (GP) models using the same three feature sets. For these comparative classifiers, we used the default parameter settings provided by the United Imaging Intelligence research platform (uAI Research Portal, uRP); their detailed hyperparameter configurations are provided in **Supplementary Table 2**.

Model performance was quantified by the AUC values of ROC curves, along with accuracy, sensitivity, and specificity. Ten-fold cross-validation was applied to assess model stability in the training set, reporting mean values and standard deviations (SDs) for performance metrics. Differences in AUC between models were assessed using DeLong’s test. Clinical utility was further examined via decision curve analysis (DCA), while integrated discrimination improvement (IDI) and net reclassification improvement (NRI) were calculated to quantify the added predictive value of the combined model. Given the inherent class imbalance in the derivation cohort, we additionally evaluated the precision-recall (PR) curve as a complementary metric to ROC analysis. Model calibration was further assessed using calibration curves with Brier scores, where lower Brier scores indicate better agreement between predicted probabilities and observed outcomes.

### 2.6 Interpretability of the models

The SHAP framework, derived from cooperative game theory, was utilized to evaluate the interpretability of the models. SHAP can quantify the contributions of individual features to the predictions of the model. Bar plots were employed when visualizing global feature importance, while beeswarm plots illustrated the direction and magnitude of the effects of features on the estimated risk. Additionally, individual-level waterfall plots were generated to demonstrate how specific features contributed to risk predictions for representative patients, thereby providing insight into personalized decision-making processes. All SHAP analyses were conducted using fastshap and shapviz in R (version 4.2.1).

### 2.7 Statistical analysis

Appropriate statistical methods were selected based on data type and distribution. Continuous variables are shown as mean ± standard deviation or median with interquartile range, and were compared using independent t-tests or Mann-Whitney U tests, depending on normality assumptions. Categorical data are reported as counts and percentages and were compared using chi-square (χ²) tests. A two-sided P < 0.05 was deemed significant, and all data were analyzed in R (version 4.2.1).

## 3 Results

### 3.1 Clinical data analysis

Two hundred and ten individuals diagnosed with HTG-AP were enrolled in the derivation cohort. Of these, 78 (37.1%) developed PPDM-A (HTG-PPDM-A group) and 132 (62.9%) did not (HTG-nPPDM-A group). Using stratified randomization, these individuals were allocated to training and testing cohorts (n = 147 and 63, respectively; ratio 7:3). The external validation cohort comprised 119 individuals, including 42 with HTG-PPDM-A and 77 without. Demographic and clinical information for both cohorts is summarized in **Supplementary Tables 3, 4**, respectively.

Comparative analysis within the training cohort (n = 147) revealed significant between-group differences in age, history of recurrence, disease severity, peritonitis incidence, SIRS, length of hospital stay, hs-CRP, and neutrophil levels (all P < 0.05). Multivariable logistic regression further identified history of recurrence (OR = 3.866,95%CI: 1.459–10.243, P = 0.007), length of hospital stay (OR = 1.205, 95%CI: 1.083–1.341, P < 0.001), and hs-CRP (OR = 1.005, 95%CI: 1.001–1.010, P = 0.019) as independent predictors of PPDM-A (**Table 1**).

**Table 1:** Training cohort characteristics.

Training cohort	HTG-AP-PPDMA (n=55)	HTG-AP-nPPDMA (n=92)	Statistical value	P value
General data				
Age, years	46.16 ± 11.469	42.27 ± 10.295	2.125^b^	0.035
Sex (male/female), n (%)	33(60.00)/22(40.00)	67(72.83)/25(27.17)	1.758^a^	0.185
Alcohol consumption, n (%)	27(49.09)	41(44.57)	0.284^a^	0.594
BMI, kg/m²	25.00(24.50,27.30)	26.45(24.42,29.18)	-1.267^c^	0.205
Smoking history, n (%)	26(47.27)	39(42.39)	0.333^a^	0.564
Fatty liver, n (%)	38(69.09)	53(57.61)	1.924^a^	0.165
Hypertension, n (%)	12(21.82)	23(25.56)	0.192^a^	0.661
Recurrent attacks, n (%)	19(34.55)	13(14.13)	8.424^a^	0.004
Biliary stones, n (%)	5(9.09)	15(16.30)	1.524^a^	0.217
Disease severity and complications				
AP Severity	20(36.36)	9(9.78)	15.358^a^	<0.001
Pleural effusion, n (%)	35(63.64)	55(59.78)	0.215^a^	0.643
Ascites, n (%)	21(38.18)	30(32.61)	0.472^a^	0.492
Peritonitis, n (%)	32(58.18)	38(41.30)	3.931^a^	0.047
SIRS, n (%)	26(47.27)	26(28.26)	5.442^a^	0.020
Length of hospital stay, days	12(9,18)	9(6,11)	-5.252^c^	<0.001
Laboratory parameters				
Blood glucose (mmol/L)	11.53(7.16,15.78)	10.96(6.56,13.97)	-1.135^c^	0.256
HDL−C(mmol/L)	0.71(0.52,0.89)	0.74(0.63,0.93)	-1.477^c^	0.140
hs−CRP(mg/L)	115.93(68.64,207.24)	69.07(24.83,128.20)	-2.829^c^	0.005
Lipase (U/L)	217.00(77.00,707.00)	248.00(87.25,922.00)	-0.739^c^	0.460
TG (mmol/L)	7.50(3.53,16.48)	8.36(4.52,16.79)	-0.416^c^	0.677
Amylase (U/L)	99.00(46.00,288.00)	142.50(54.00,366.95)	-0.643^c^	0.521
LDL−C(mmol/L)	2.513 ± 1.281	2.609 ± 1.363	1.146^b^	0.674
WBC count (×10^9^/L)	13.66(10.58,16.39)	12.81(9.40,15.76)	-1.759^c^	0.079
Neutrophil percentage, %	3.982 ± 0.866	3.539 ± 0.519	-3.549^b^	<0.001
Serum calcium (mmol/L)	2.39(2.22,2.49)	2.31(2.19,2.44)	-0.422^c^	0.254
Total cholesterol (mmol/L)	6.63(4.63,7.83)	6.62(4.99,8.16)	-0.290^c^	0.772

a—χ^2^ value; b—t value; c—Z value, a Data are expressed as number (%). Group comparisons were performed using the χ^2^-test or Fisher’s exact test, as appropriate. b Data are expressed as mean ± standard deviation. Group comparisons were performed using the independent samples t test. c Data are expressed as median (interquartile range). Group comparisons were performed using the Mann-Whitney U-test. P < 0.05 was considered statistically significant.

### 3.2 Selection of features and determination of rad-scores

**Figure 3:**
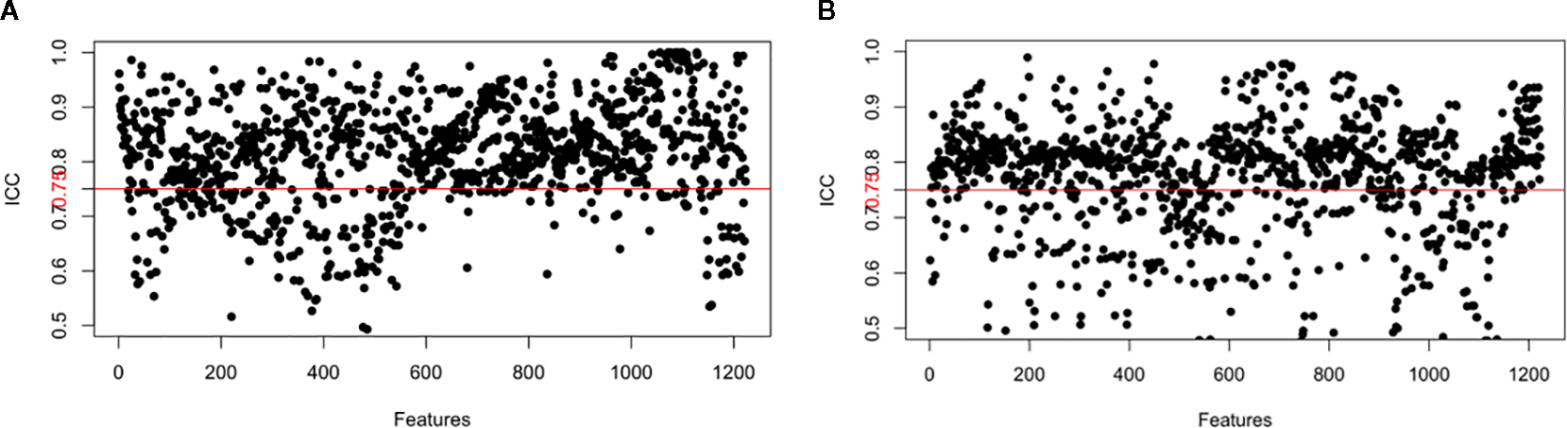
Scatter plots of ICCs for evaluating the reproducibility of radiomic features.The plots illustrate the inter-observer agreement of the extracted high-dimensional features. **(A)** Features derived from the T2WI sequence, with 950 features retained. **(B)** Features derived from the CE-T1WI sequence, with 840 features retained. The red horizontal solid line indicates the predetermined reliability threshold of ICC = 0.75. Features plotted above this line were considered highly reproducible and were selected for subsequent dimensionality reduction. ICC, intraclass correlation coefficient; T2WI, T2-weighted imaging; CE-T1WI, contrast-enhanced T1-weighted imaging.

**Figure 4:**
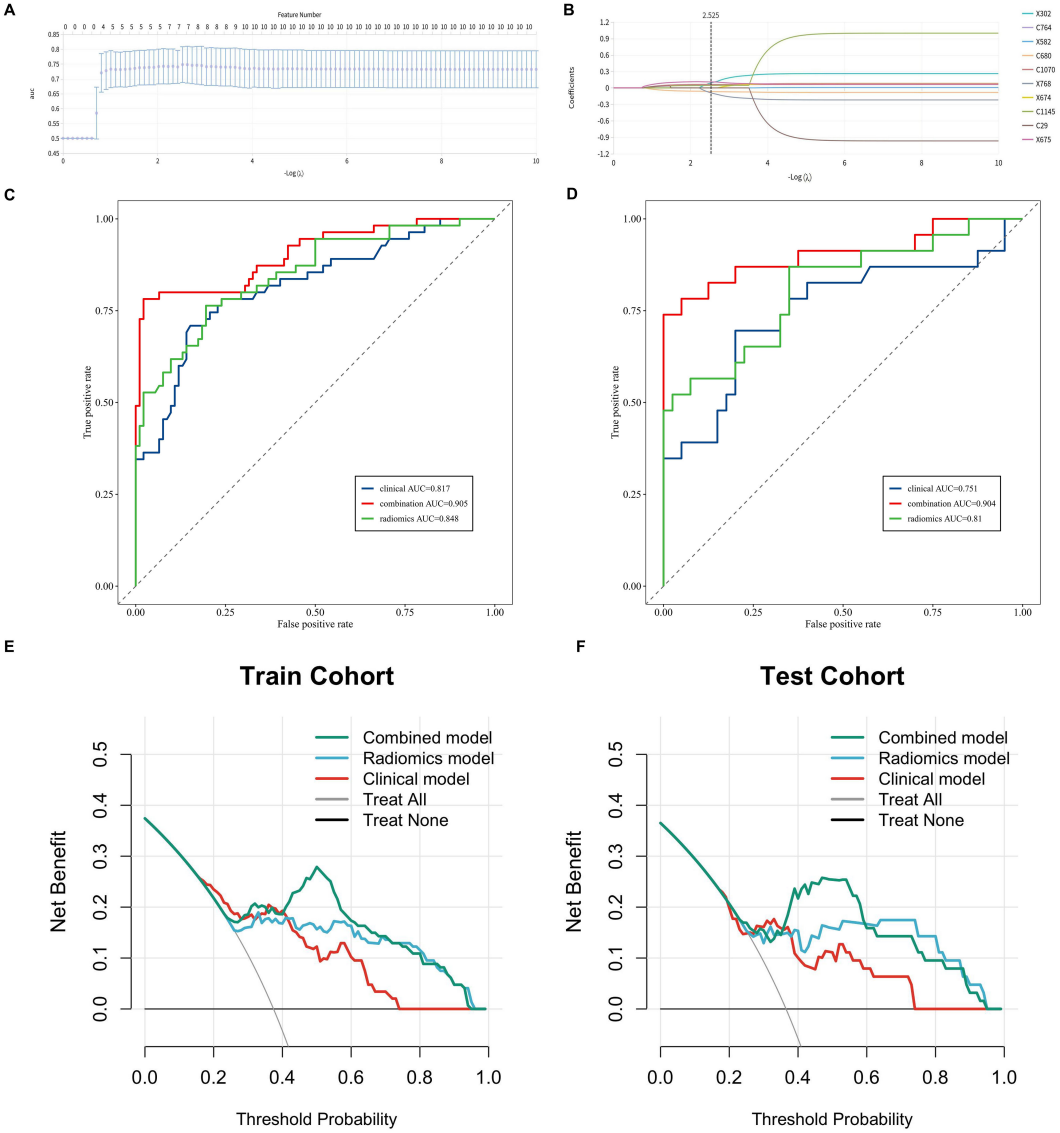
Feature selection and predictive performance evaluation of the machine learning models. **(A)** Tuning parameter selection in the LASSO model using 10-fold cross-validation. The y-axis represents the AUC. **(B)** LASSO coefficient profiles of the selected radiomic features. The vertical dashed line is drawn at the optimal regularization parameter (−log(λ) = 2.525), which minimizes the cross-validation error. **(C, D)** ROC curves comparing the discrimination performance of the clinical, radiomics, and combined models in the training cohort **(C)** and the internal testing cohort **(D)**. **(E, F)** DCA evaluating the clinical utility of the three models in the training cohort **(E)** and the internal testing cohort **(F)**. The combined model consistently demonstrates a higher net clinical benefit across the threshold probability range of 0.2 to 0.8 compared to single-modality models and default strategies. LASSO, least absolute shrinkage and selection operator; AUC, area under the curve; ROC, receiver operating characteristic; DCA, decision curve analysis.

After assessing feature reproducibility using ICC criteria (**Figures 3A, B**), 950 features from the T2WI sequence and 840 features from the CE-T1WI sequence were retained as robust candidate features. Subsequent dimensionality reduction involved a multistep filtering strategy. Features with variance < 1.0 were removed. Next, the SelectKBest method was utilized to retain features that showed substantial differences between the groups (P < 0.05). Finally, LASSO regression further refined the feature set and performed redundancy minimization. The optimal regularization parameter was identified at −log(λ) = 2.525 (**Figures 4A, B**).

The sequential selection pipeline ultimately identified seven key radiomic features, including three derived from T2WI and four from CE-T1WI. These consisted of one log-sigma feature and six wavelet-transformed features. For each patient, a composite Rad-score was computed as a weighted linear combination of these selected features and their respective non-zero LASSO coefficients (**Table 2**), with an intercept of 0.374. This Rad-score, reflecting subtle microstructural alterations in pancreatic tissue, was subsequently incorporated as a core variable in the combined predictive model.

**Table 2:** The seven selected radiomic features and their corresponding non-zero coefficients derived from the LASSO regression model.

Image type	Feature class	Feature name	Coefficient	Feature ID
wavelet-LHH	firstorder	Median	0.108	X675
log-sigma-1-5-mm-3D	firstorder	Mean	0.090	X302
wavelet-HHH	glcm	Imc2	0.063	C1070
wavelet-HLL	firstorder	Kurtosis	0.056	C764
wavelet-LLL	firstorder	Skewness	0.044	C1145
wavelet-LHH	firstorder	Skewness	-0.067	C680
wavelet-HLL	firstorder	Median	-0.090	X768

LASSO, least absolute shrinkage and selection operator; glcm, gray-level co-occurrence matrix; CE-T1WI, contrast-enhanced T1-weighted imaging; T2WI, T2-weighted imaging. In the Feature ID column, the prefixes ‘C’ and ‘X’ denote features extracted from the CE-T1WI and T2WI sequences, respectively. For each patient, the composite Rad-score is calculated by summing the products of the feature values and their respective coefficients, alongside a constant intercept of 0.374.

### 3.3 SHAP analysis of radiomics features

**Figure 5:**
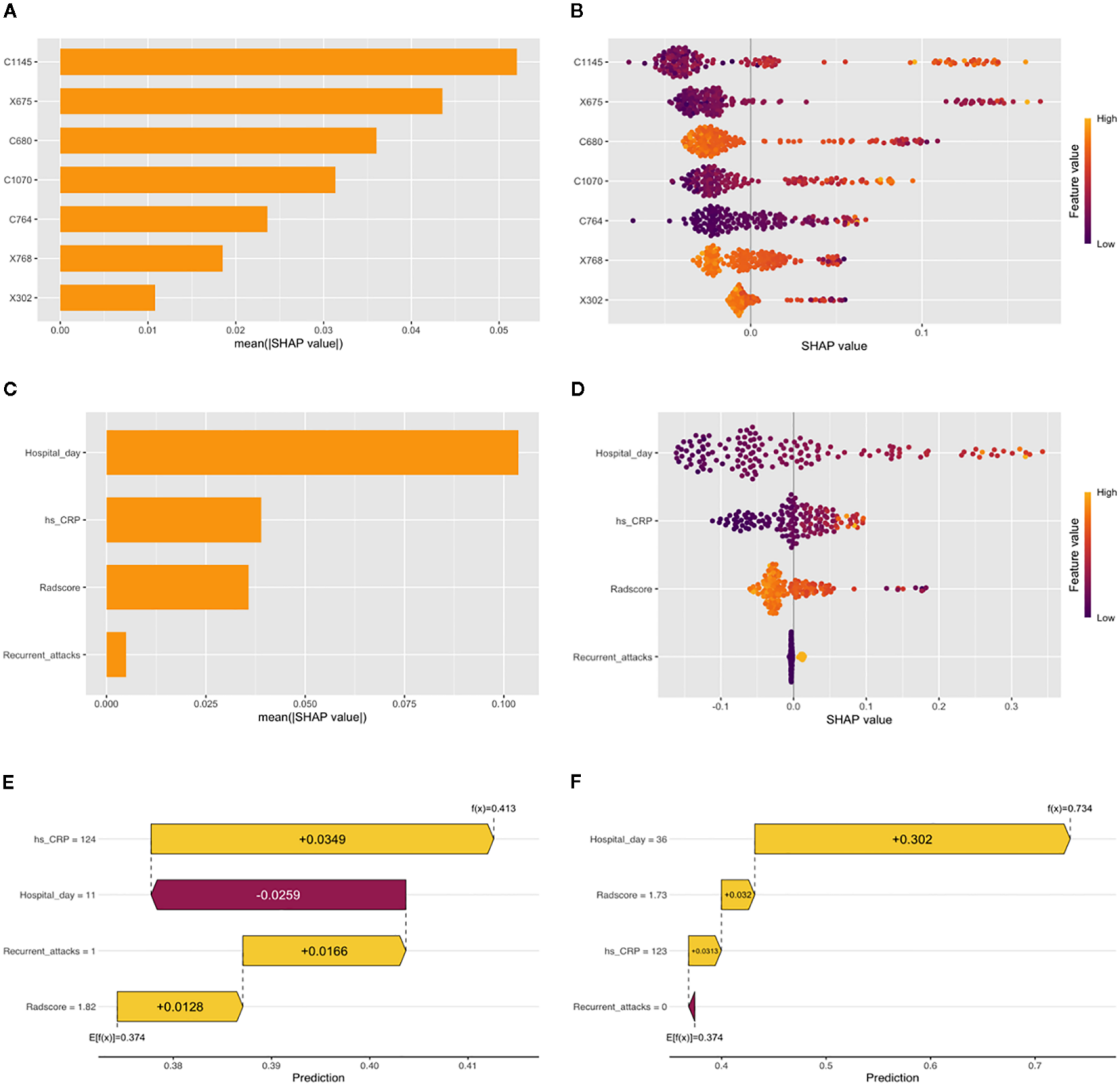
Global and local interpretability of the predictive models using SHapley Additive exPlanations (SHAP) analysis. **(A)** Global feature importance ranking of the radiomics-only model, displaying the mean absolute SHAP values for the top features. **(B)** SHAP summary beeswarm plot for the radiomics-only model, illustrating the non-linear relationship between feature values (color-coded from low to high) and their impact on model predictions. **(C, D)** Global feature importance **(C)** and summary beeswarm plot **(D)** for the combined model, identifying the length of hospital stay (Hospital_day), hs-CRP, Rad-score, and recurrent attacks as the key predictors. **(E, F)** Local SHAP waterfall plots for two representative patients. **(E)** Predicted risk: 41.3%. A short hospital stay (11 days, −0.0259) was counteracted by a high Rad-score (1.82, + 0.0128), recurrent attacks (+0.0166), and elevated hs−CRP (124 mg/L, +0.0349). **(F)** Predicted risk: 73.4%. A prolonged hospital stay (36 days, +0.302), high Rad−score (1.73, + 0.0320), and elevated hs−CRP (123 mg/L, +0.0313) drove the risk increase.

To elucidate the contribution of individual radiomic features within a non-linear modeling framework, SHAP evaluation was undertaken on the radiomics-only model. Global feature importance ranking indicated that features C1145 and X675 had the greatest influence on model predictions (**Figure 5A**). Notably, this ranking differed slightly from the linear coefficients derived from LASSO, underscoring the ability of tree-based algorithms to capture higher-order interactions among features. The SHAP summary beeswarm plot (**Figure 5B**) further illustrated the complex, non-linear relationships between feature values and predicted risk. This visualization provided intuitive evidence demonstrating that radiomic features derived from dual MRI sequences offer complementary pathological information.

### 3.4 Model performance evaluation

Three predictive models based on RF algorithms were developed: a clinical model, a radiomics model, and a combined model. In the internal training cohort, the combined model achieved the highest discrimination with an AUC of 0.905 (95%CI: 0.847–0.954), outperforming both the radiomics (AUC = 0.848 [95%CI: 0.783–0.914]) and clinical (AUC = 0.817 [95%CI: 0.734–0.890]) models. Consistent results were found in the internal testing cohort, where the corresponding AUCs were 0.904 (95%CI: 0.803–0.984), 0.810 (95%CI: 0.693–0.927), and 0.751 (95%CI: 0.612–0.889), respectively (**Figures 4C, D**). In addition to superior discrimination, the combined model demonstrated high sensitivity, specificity, and overall accuracy across both datasets (**Table 3**).

**Table 3:** Predictive performance of the combined, radiomics, and clinical models across the training and test sets.

Cohort	Model	AUC(95%CI)	Specificity	Accuracy	Sensitivity
Training set	Combined model	0.905 (95%CI: 0.847–0.954)	0.978 (95%CI: 0.916–0.996)	0.905 (95%CI: 0.842–0.945)	0.782 (95%CI: 0.646–0.878)
	Radiomics model	0.848 (95%CI: 0.783–0.914)	0.902 (95%CI: 0.818–0.952)	0.782 (95%CI: 0.705–0.844)	0.581 (95%CI: 0.441–0.711)
	Clinical model	0.817 (95%CI: 0.734–0.890)	0.924 (95%CI: 0.844–0.966)	0.748 (95% CI: 0.669–0.815)	0.455 (95%CI: 0.322–0.593)
Test set	Combined model	0.904 (95%CI: 0.803–0.984)	0.975 (95%CI: 0.853–0.999)	0.889 (95%CI: 0.778–0.950)	0.739 (95%CI: 0.513–0.889)
	Radiomics model	0.810 (95%CI: 0.693–0.927)	0.950 (95%CI: 0.820–0.991)	0.794 (95%CI: 0.670–0.881)	0.522 (95%CI: 0.311–0.726)
	Clinical model	0.751 (95%CI: 0.612–0.889)	0.950 (95%CI: 0.818–0.991)	0.746 (95%CI: 0.618–0.844)	0.391 (95%CI: 0.205–0.612)

AUC, area under the receiver operating characteristic curve; CI, confidence interval. Performance metrics are presented as the point estimate followed by the 95% confidence interval in parentheses. The combined model demonstrated superior discriminative ability compared to the single-modality models in both cohorts.

To complement the ROC analysis under class imbalance, we constructed PR curves for the combined model. The PR-AUC values were 0.898 (95% CI: 0.830–0.950) in the training cohort and 0.907 (95% CI: 0.800–0.980) in the testing cohort, indicating that the model maintained robust discriminative ability despite the class imbalance in the dataset (**Supplementary Figure 2**).

Calibration curves with Brier scores were used to evaluate model performance. The Brier scores for the training and testing cohorts were 0.106 and 0.099, respectively, reflecting favorable alignment between predicted and observed outcomes. The corresponding intercepts and slopes were 0.053, 0.251 and 1.392, 1.638, respectively (**Supplementary Figure 3**).

Model stability was further evaluated via ten-fold cross-validation within the training cohort, yielding a mean AUC of 0.895 ± 0.074 (CV = 8.30%), with a stable distribution range (0.758-0.963) (**Supplementary Figure 4**).

DeLong’s test revealed that the combined model significantly outperformed the clinical model in both the training (P = 0.005) and testing (P = 0.044) cohorts. In comparison to the radiomics model, the combined model exhibited a significant advantage in the internal training cohort (P = 0.021), although the difference was non-significant in the internal testing cohort (P = 0.113).

To further validate the choice of RF as the primary classifier, we compared its performance against SVM, LR, and GP using the combined feature set. In the training cohort, RF achieved the highest AUC (0.905), outperforming GP (0.871), SVM (0.856), and LR (0.856). This pattern was consistent in the testing cohort, where RF again demonstrated superior discrimination (AUC 0.904) compared with LR (0.851), GP (0.836), and SVM (0.830). Detailed performance metrics for all classifiers are provided in **Supplementary Table 5**.

DCA demonstrated consistently higher net clinical benefit in the combined model compared to either single-modality model across various threshold probabilities in both cohorts (**Figures 4E, F**). The combined model was also superior to the default “treat-all” and “treat-none” approaches within the clinically relevant threshold of 0.2–0.8.

Risk reclassification metrics further supported the superiority of the combined model (**Table 4**). In the training cohort, the combined model significantly improved classification accuracy compared with the clinical model, as reflected by categorical NRI (0.371), continuous NRI (0.354), and IDI (0.121) (all P < 0.05). Improvements were also observed relative to the radiomics model (categorical NRI = 0.265, IDI = 0.039; all P < 0.001). In the internal testing cohort, the combined model still exhibited significant gains over the clinical model in categorical NRI (0.373, P = 0.003) and IDI (0.147, P < 0.001), although the continuous NRI was not statistically significant. Compared with the radiomics model, only the categorical NRI remained significant (P = 0.007).

**Table 4:** Risk reclassification evaluation comparing the combined model with the clinical and radiomics models.

Cohort	Model comparison	Categorical NRI (95% CI)	P value	Continuous NRI (95% CI)	P value	IDI (95% CI)	P value
Train	Clinical vs Combined	0.371 (0.224–0.517)	<0.001	0.354 (0.071–0.637)	0.014	0.121 (0.072–0.169)	<0.001
Train	Radiomics vs Combined	0.265 (0.148–0.382)	<0.001	0.323 (-0.007–0.653)	0.055	0.039 (0.017–0.061)	<0.001
Test	Clinical vs Combined	0.373 (0.129–0.617)	0.003	0.215 (-0.230–0.660)	0.343	0.147 (0.066–0.227)	<0.001
Test	Radiomics vs Combined	0.242 (0.067–0.418)	0.007	0.107 (-0.404–0.617)	0.683	0.024 (-0.027–0.074)	0.359

NRI, net reclassification improvement; IDI, integrated discrimination improvement; CI, confidence interval. Categorical NRI, continuous NRI, and IDI were calculated to quantify the added predictive value of the combined model over the single-modality models. Positive values indicate an improvement in the predictive accuracy of the combined model compared to the reference model (Clinical or Radiomics). A p-value < 0.05 was considered statistically significant.

### 3.5 Mechanistic insights and individualized prediction using the combined model

SHAP analysis of the combined model (**Figures 5C, D**) identified length of hospital stay, hs-CRP level, Rad-score, and history of AP recurrence as the four most influential predictors of HTG-PPDM-A risk. To illustrate patient−specific stratification, we generated individual SHAP waterfall plots against a cohort−wide baseline risk of 37.4%. In Case 1 (**Figure 5E**; predicted risk: 41.3%), a short hospital stay of 11 days lowered the risk (SHAP: -0.0259), but this effect was offset by a high Rad−score of 1.82 (+0.0128), history of AP recurrence (Recurrent_attacks = 1, + 0.0166), and an elevated hs−CRP level of 124 mg/L (+0.0349). In contrast, in Case 2 (**Figure 5F**; predicted risk: 73.4%), a prolonged hospital stay of 36 days dominated the risk increase (+0.302), further reinforced by a Rad−score of 1.73 (+0.0320) and an hs−CRP level of 123 mg/L (+0.0313). These cases demonstrate the model’s clinical utility in dynamically weighting both macro−clinical and micro−radiomic parameters.

### 3.6 External validation

**Figure 6:**
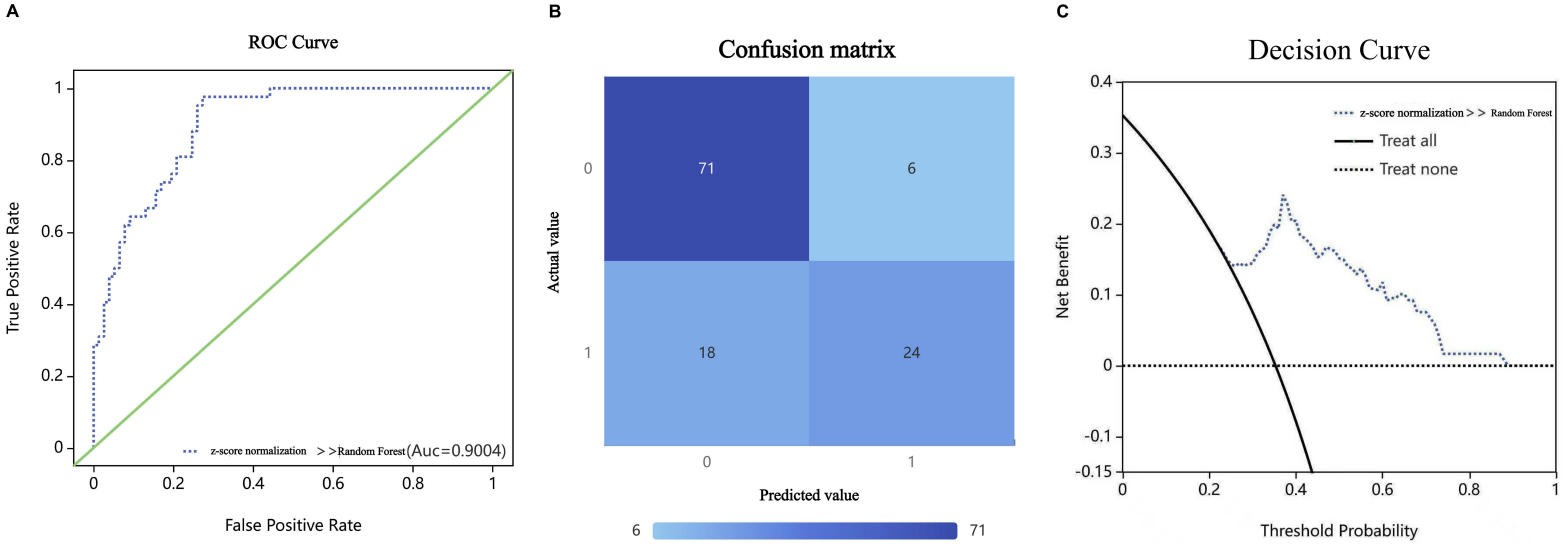
Predictive performance and clinical utility of the combined model in the independent external validation cohort. **(A)** ROC curve demonstrating the robust discriminative ability of the model with an AUC of 0.900. **(B)** Confusion matrix illustrating the detailed classification results. The confusion matrix demonstrated that 95 of 119 patients were correctly classified, including 71 true negatives and 24 true positives. However, notably, the model exhibited a relatively high false-negative rate, misclassifying 18 out of 42 true cases as low-risk, while maintaining a low false-positive count (n = 6).while **(C)** DCA indicating the clinical applicability of the combined model. The model yields a greater net clinical benefit compared to the default “treat-all” (solid black line) and “treat-none” (dotted horizontal line) strategies across a broad range of threshold probabilities. AUC, area under the curve; ROC, receiver operating characteristic; DCA, decision curve analysis.

The combined model retained its discriminative capability when tested in the independent validation cohort, achieving an AUC of 0.900 (95% CI: 0.848-0.953) (**Figure 6A**). Additional performance metrics included an accuracy of 0.798, an F1 score of 0.667, a precision of 0.800, a sensitivity of 0.571, and a specificity of 0.922, further supporting its reliability.

The confusion matrix (**Figure 6B**) demonstrated that 95 of 119 patients were correctly classified, including 71 true negatives and 24 true positives. However, notably, the model exhibited a relatively high false-negative rate, misclassifying 18 out of 42 true HTG-PPDM-A cases as low-risk, while maintaining a low false-positive count (n = 6). DCA (**Figure 6C**) indicated that the combined model consistently delivered greater net clinical benefit than the “treat-all” or “treat-none” approaches across a threshold probability range from 0.25 to 0.75. For the external validation cohort, the Brier score was 0.132, with an intercept of 0.051 and a slope of 1.340 (**Supplementary Figure 3**). Collectively, these findings confirm the model’s moderate generalizability and suggest its potential clinical utility.

## 4 Discussion

In the present study, a non-invasive predictive framework that integrates dual-sequence MRI radiomics (T2WI and CE-T1WI) with clinical variables was established and validated as a reliable prognostic tool for estimating the risk of HTG-PPDM-A prior to discharge (5). The combined model showed consistently high discriminative ability in all three cohorts, with AUC values of 0.905, 0.904, and 0.900 for the training, internal testing, and external validation sets, respectively. Incorporation of the SHAP interpretability framework further enabled visualization of feature contributions at both the individual and population levels, thereby enhancing model transparency and clinical applicability for personalized outpatient follow-up and timely preventive interventions.

Prior investigations of PPDM-A risk have largely relied on laboratory markers, conventional clinical indicators, or single-modality imaging approaches. However, predictive models based solely on fasting glucose or isolated inflammatory biomarkers show limited ability to capture pancreatic endocrine dysfunction (27–29). Although multivariable clinical nomograms have improved predictive accuracy to some extent, they primarily reflect systemic metabolic disturbances and fail to adequately quantify irreversible injury within the pancreatic microenvironment, which is a key driver of PPDM-A pathogenesis (30). To address these limitations, quantitative imaging strategies have been increasingly explored for pancreatic assessment. While preliminary efforts demonstrated the feasibility of contrast-enhanced CT-based radiomics in characterizing tissue injury (31), recent advancements in magnetic resonance imaging have further established the utility of single-sequence T2WI radiomics for characterizing acute-phase inflammatory severity (32). However, predicting long-term endocrine sequelae such as PPDM-A requires capturing more intricate microvascular and parenchymal changes. The distinct pathophysiological mechanisms underlying HTG-AP, including early microvascular thrombosis and FFA-mediated lipotoxicity, necessitate more sensitive imaging strategies (8, 33). Compared with CT, MRI provides superior sensitivity for detecting subtle interstitial edema and microcirculatory disturbances while avoiding ionizing radiation. Specifically, T2WI is highly responsive to early fluid accumulation, whereas late arterial phase CE-T1WI enables detailed assessment of microvascular perfusion surrounding pancreatic islets (12, 13). By extending beyond single−sequence MRI protocols, the current dual−sequence approach successfully converts subtle, multi−dimensional spatial heterogeneity into quantitative features (15, 34), offering a robust, radiation-free approach for tracking early microstructural alterations in the pancreas.

Among machine learning classifiers, we selected RF as the primary modeling algorithm after benchmarking against SVM, LR, and GP using the same feature sets. The superior performance of RF in our dataset may be attributed to its inherent robustness to high-dimensional, correlated radiomic features and its ability to capture non-linear interactions without requiring extensive hyperparameter tuning. These characteristics make RF particularly well-suited for integrating heterogeneous radiomic and clinical data. The Rad-score developed in this study was derived from seven key radiomic features, including three from T2WI and four from CE-T1WI, underscoring the complementary nature of these imaging sequences. To address the interpretability challenges commonly associated with machine learning, a hierarchical SHAP interpretation framework was employed to elucidate the internal logic of feature-driven decisions and clarify their pathophysiological relevance. In the radiomics-only model, wavelet-derived features from both sequences were identified as dominant contributors. For instance, LoG-based features from T2WI likely capture subtle disruptions at the interface between inflammatory exudates and normal acinar structures (15, 32). In addition, elevated higher-order features from CE-T1WI reflect marked heterogeneity in contrast distribution, consistent with microvascular obstruction and perfusion deficits induced by lipotoxic injury (35). Interestingly, certain features such as C1145 (wavelet-LLL_firstorder_Skewness) exhibited relatively low weights in the linear LASSO model but became highly influential in the non-linear SHAP framework. From a biophysical perspective, skewness quantifies asymmetry in voxel intensity distribution after noise filtering (36), which aligns with the heterogeneous pathological landscape of HTG-AP, characterized by focal ischemic necrosis coexisting with compensatory hyperemia (33). This non-linear reversal of feature importance suggests that the synergistic interaction between edema and micro-perfusion abnormalities, rather than either factor alone, may accelerate β-cell dysfunction and exhaustion (8, 33). Consequently, these radiomic features represent meaningful biomarkers that reflect the cascade of ischemia, hypoxia, and apoptosis within pancreatic islets.

At the clinical level, SHAP-based global analysis of the combined model identified length of hospital stay, hs-CRP level, Rad-score, and history of AP recurrence as the most influential predictors of HTG-PPDM-A. This hierarchy is consistent with the biological progression of the disease. Prolonged hospitalization often indicates more severe local injury or delayed systemic recovery, both of which are associated with sustained metabolic stress and increased long-term risk (37). Elevated hs-CRP levels reflect a pronounced inflammatory response driven by HTG-induced SIRS, where pro-inflammatory mediators disrupt islet integrity and exacerbate insulin resistance (38). Additionally, recurrent episodes of pancreatitis represent cumulative insults that promote progressive parenchymal destruction and fibrosis, ultimately depleting endocrine reserve capacity (39). Importantly, patient-level SHAP visualizations highlighted the complementary roles of radiomic and clinical features in individualized prediction. In patients with clinically occult or subclinical presentations and short hospital stays, who might otherwise be misclassified as low-risk via routine assessments, the model successfully identified elevated risk based on high Rad-scores and increased hs-CRP levels. This dynamic allocation mechanism of multidimensional risk weights not only elucidates the source of the model’s predictive efficacy but also provides evidence-based support for personalized follow-up and management strategies.

When translating our predictive framework into clinical workflows, the model’s sensitivity in the external validation cohort (0.571) warrants careful consideration. While the high specificity (0.922) ensures that predicted high-risk patients are highly likely to benefit from early intervention, the model missed 18 out of 42 true PPDM−A cases. Pathophysiologically, this sub−optimal sensitivity may reflect the progressive nature of post−pancreatitis β−cell exhaustion, as the decline in islet function following acute pancreatic injury often occurs gradually rather than as an acute event (40). Islet microvascular damage and subsequent endocrine decline often occur gradually; thus, pre−discharge MRI radiomics and baseline clinical indicators may not fully capture the latent metabolic dysfunction that only manifests at 3 months post−discharge.

To mitigate the risk of under−monitoring, our model should not serve as an absolute binary gatekeeper for clinical surveillance. Instead, we propose a two−tiered clinical risk−management strategy: patients flagged as high−risk should undergo immediate, intensive lifestyle counseling and active dynamic glucose monitoring, whereas patients classified as low−risk must still undergo routine outpatient glycemic screening (e.g., HbA1c or OGTT) if clinically indicated.

Several limitations should be noted. First, potential selection and attrition biases are inherent to our retrospective design, primarily stemming from the exclusion of patients with poor MRI image quality. Second, baseline dynamic metabolic parameters—such as homeostasis model assessment of insulin resistance (HOMA−IR), beta−cell function (HOMA−β), or longitudinal pre−admission glycemic trajectories—were unavailable in our retrospective database. Unmeasured post−discharge confounding factors, including lifestyle modifications and compliance with lipid−lowering therapies, may also influence the clinical development of PPDM−A. Third, although validated using a geographically distinct cohort, both datasets were nested within a single medical system, which may limit the immediate generalizability of our model across different scanner vendors. Future prospective, multi−center registry studies with standardized scanning protocols and longitudinal metabolic profiling are warranted to further validate and refine the clinical utility of our predictive framework.

## 5 Conclusion

In conclusion, we developed and validated a machine learning-based predictive model that integrates dual-sequence MRI radiomics with clinical variables for the non-invasive, pre-discharge prediction of long-term PPDM-A risk in patients with HTG-AP. This prognostic tool holds the potential to facilitate personalized outpatient follow-up and timely preventive interventions for patients recovering from HTG-AP.

## Data Availability

The raw data supporting the conclusions of this article will be made available by the authors, without undue reservation.

## References

[1] Trikudanathan G Yazici C Evans Phillips A Forsmark CE . Diagnosis and management of acute pancreatitis. Gastroenterology. (2024) 167:673–88. doi: 10.1053/j.gastro.2024.02.052 38759844

[2] Zahariev OJ Bunduc S Kovács A Demeter D Havelda L Budai BC . Risk factors for diabetes mellitus after acute pancreatitis: A systematic review and meta-analysis. Front Med (Lausanne). (2023) 10:1257222. doi: 10.3389/fmed.2023.1257222 38264039 PMC10803425

[3] Petrov MS . Diabetes of the exocrine pancreas: American Diabetes Association-compliant lexicon. Pancreatology. (2017) 17:523–6. doi: 10.1016/j.pan.2017.06.007 28655595

[4] Olesen SS Viggers R Drewes AM Vestergaard P Jensen MH . Risk of major adverse cardiovascular events, severe hypoglycemia, and all-cause mortality in postpancreatitis diabetes mellitus versus type 2 diabetes: A nationwide population-based cohort study. Diabetes Care. (2022) 45:1326–34. doi: 10.2337/dc21-2531 35312752

[5] Petrov MS . Diagnosis of endocrine disease: Post-pancreatitis diabetes mellitus: Prime time for secondary disease. Eur J Endocrinol. (2021) 184:R137–49. doi: 10.1530/eje-20-0468 33460393

[6] American Diabetes Association Professional Practice Committee . Classification and diagnosis of diabetes: Standards of medical care in diabetes-2022. Diabetes Care. (2022) 45:S17–38. doi: 10.2337/dc22-S002 34964875

[7] Fan Z Zhang Y Li J He W Bai X Cai Y . Global burden and characterization of hypertriglyceridemia-induced acute pancreatitis: Results from a systematic review and a multi-center cohort study. Sci China Life Sci. (2025) 68:3010–20. doi: 10.1007/s11427-024-2900-6 40550999

[8] Kiss L Fűr G Pisipati S Rajalingamgari P Ewald N Singh V . Mechanisms linking hypertriglyceridemia to acute pancreatitis. Acta Physiol (Oxf). (2023) 237:e13916. doi: 10.1111/apha.13916 36599412

[9] Fang C Ding Y Wang X Teng X . Metabolic disturbances in acute pancreatitis: Mechanisms and therapeutic implications. Front Endocrinol (Lausanne). (2025) 16:1579457. doi: 10.3389/fendo.2025.1579457 40937420 PMC12420230

[10] Yang DD Gao J Liu J Liu C Liang Y . Patients-associated compound etiology may have more severe acute pancreatitis: A retrospective cohort study. Quant Imaging Med Surg. (2022) 12:4109–19. doi: 10.21037/qims-21-1157 35919042 PMC9338379

[11] Hu R Yang H Zeng GF Wang ZG Zhou D Luo YD . A radiomics model of contrast-enhanced computed tomography for predicting post-acute pancreatitis diabetes mellitus. Quant Imaging Med Surg. (2024) 14:2267–79. doi: 10.21037/qims-23-1232 38545039 PMC10963832

[12] Tartari C Porões F Schmidt S Abler D Vetterli T Depeursinge A . Mri and ct radiomics for the diagnosis of acute pancreatitis. Eur J Radiol Open. (2025) 14:100636. doi: 10.1016/j.ejro.2025.100636 39967811 PMC11833635

[13] Jiang ZQ Xiao B Zhang XM Xu HB . Early-phase vascular involvement is associated with acute pancreatitis severity: A magnetic resonance imaging study. Quant Imaging Med Surg. (2021) 11:1909–20. doi: 10.21037/qims-20-280 33936974 PMC8047341

[14] Tang W Zhang XM Xiao B Zeng NL Pan HS Feng ZS . Magnetic resonance imaging versus acute physiology and chronic healthy evaluation ii score in predicting the severity of acute pancreatitis. Eur J Radiol. (2011) 80:637–42. doi: 10.1016/j.ejrad.2010.08.020 20843620

[15] Lambin P Leijenaar RTH Deist TM Peerlings J de Jong EEC van Timmeren J . Radiomics: The bridge between medical imaging and personalized medicine. Nat Rev Clin Oncol. (2017) 14:749–62. doi: 10.1038/nrclinonc.2017.141 28975929

[16] Zhang J Lv Y Hou J Zhang C Yua X Wang Y . Machine learning for post-acute pancreatitis diabetes mellitus prediction and personalized treatment recommendations. Sci Rep. (2023) 13:4857. doi: 10.1038/s41598-023-31947-4 36964219 PMC10038980

[17] Hu R Chen YL Li GJ Luo YD Zhou D Wang ZG . An interpretable radiomics model based on contrast-enhanced pancreatic computed tomography for predicting the prognosis of post-acute pancreatitis diabetes mellitus. BMC Med Imaging. (2026) 26:201. doi: 10.1186/s12880-026-02258-7 41820881 PMC13097733

[18] Tjoa E Guan C . A survey on explainable artificial intelligence (xai): Toward medical xai. IEEE Trans Neural Netw Learn Syst. (2021) 32:4793–813. doi: 10.1109/tnnls.2020.3027314 33079674

[19] von Elm E Altman DG Egger M Pocock SJ Gøtzsche PC Vandenbroucke JP . The strengthening the reporting of observational studies in epidemiology (strobe) statement: Guidelines for reporting observational studies. Lancet. (2007) 370:1453–7. doi: 10.1016/s0140-6736(07)61602-x 18064739

[20] Hamesch K Hollenbach M Guilabert L Lahmer T Koch A . Practical management of severe acute pancreatitis. Eur J Intern Med. (2025) 133:1–13. doi: 10.1016/j.ejim.2024.10.030 39613703

[21] Virani SS Morris PB Agarwala A Ballantyne CM Birtcher KK Kris-Etherton PM . 2021 acc expert consensus decision pathway on the management of ascvd risk reduction in patients with persistent hypertriglyceridemia: A report of the American College of Cardiology Solution Set Oversight Committee. J Am Coll Cardiol. (2021) 78:960–93. doi: 10.1016/j.jacc.2021.06.011 34332805

[22] Ding L Li S Cao L Wang L Zhou J Mao W . Recurrence of hypertriglyceridemia-associated acute pancreatitis: A multicenter, prospective cohort study. Eur J Intern Med. (2024) 125:98–103. doi: 10.1016/j.ejim.2024.03.022 38538416

[23] American Diabetes Association Professional Practice Committee . Diagnosis and classification of diabetes: Standards of care in diabetes-2024. Diabetes Care. (2024) 47:S20–42. doi: 10.2337/dc24-S002 38078589 PMC10725812

[24] Qian S Liu Y Wang W Ni W Zhao S Xu Y . Risk stratification and predictive value of glucose variability for the development of post-acute pancreatitis diabetes mellitus. Front Endocrinol (Lausanne). (2025) 16:1501530. doi: 10.3389/fendo.2025.1501530 41404504 PMC12702752

[25] Zwanenburg A Vallières M Abdalah MA Aerts H Andrearczyk V Apte A . The image biomarker standardization initiative: Standardized quantitative radiomics for high-throughput image-based phenotyping. Radiology. (2020) 295:328–38. doi: 10.1148/radiol.2020191145 32154773 PMC7193906

[26] Koo TK Li MY . A guideline of selecting and reporting intraclass correlation coefficients for reliability research. J Chiropr Med. (2016) 15:155–63. doi: 10.1016/j.jcm.2016.02.012 27330520 PMC4913118

[27] Jeon C Hart PA Li L Yang Y Chang E Bellin MD . Development of a clinical prediction model for diabetes in chronic pancreatitis: The predict3c study. Diabetes Care. (2023) 46:46–55. doi: 10.2337/dc22-1414 36382801 PMC9797648

[28] Guo SY Yang HY Ning XY Guo WW Chen XW Xiong M . Combination of body mass index and fasting blood glucose improved predictive value of new-onset prediabetes or diabetes after acute pancreatitis: A retrospective cohort study. Pancreas. (2022) 51:388–93. doi: 10.1097/mpa.0000000000002025 35695791 PMC9257058

[29] Ginzburg G Debnath P Zhang Y Ata NA Farrell PR Garlapally V . Clinical and imaging predictors for the development of diabetes mellitus following a single episode of acute pancreatitis in youth. Dig Liver Dis. (2025) 57:519–25. doi: 10.1016/j.dld.2024.10.009 39462712 PMC11769733

[30] Tang LL Zhang Q Song SY Liu N Du QL Zhong ST . Individualized prediction of post-acute pancreatitis diabetes mellitus by combining lipid metabolism and anatomical features. Insights Imaging. (2025) 16:161. doi: 10.1186/s13244-025-02039-w 40745504 PMC12314159

[31] Wan X Wang Y Liu Z Liu Z Zhong S Huang X . Development of an interpretable machine learning model based on ct radiomics for the prediction of post acute pancreatitis diabetes mellitus. Sci Rep. (2025) 15:1985. doi: 10.1038/s41598-025-86290-7 39814953 PMC11736066

[32] Li Y Wang Y Wan X Huang X . Radiomics derived from MRI T2-weighted imaging combined with clinical variables for predicting disease severity in hypertriglyceridemic acute pancreatitis. Abdom Radiol (NY). (2026). doi: 10.1007/s00261-026-05487-0 41925844

[33] Qiu M Zhou X Zippi M Goyal H Basharat Z Jagielski M . Comprehensive review on the pathogenesis of hypertriglyceridaemia-associated acute pancreatitis. Ann Med. (2023) 55:2265939. doi: 10.1080/07853890.2023.2265939 37813108 PMC10563627

[34] Mayerhoefer ME Materka A Langs G Häggström I Szczypiński P Gibbs P . Introduction to radiomics. J Nucl Med. (2020) 61:488–95. doi: 10.2967/jnumed.118.222893 32060219 PMC9374044

[35] Hu R Yang H Chen Y Zhou T Zhang J Chen TW . Dynamic contrast-enhanced mri for measuring pancreatic perfusion in acute pancreatitis: A preliminary study. Acad Radiol. (2019) 26:1641–9. doi: 10.1016/j.acra.2019.02.007 30885415

[36] van Griethuysen JJM Fedorov A Parmar C Hosny A Aucoin N Narayan V . Computational radiomics system to decode the radiographic phenotype. Cancer Res. (2017) 77:e104–7. doi: 10.1158/0008-5472.Can-17-0339 29092951 PMC5672828

[37] Pichardo-Lowden A Goodarzi MO Trikudanathan G Serrano J Dungan KM . Risk and factors determining diabetes after mild, nonnecrotizing acute pancreatitis. Curr Opin Gastroenterol. (2024) 40:396–403. doi: 10.1097/mog.0000000000001055 38935336 PMC11305911

[38] Blom SE Narsian PR Behan-Bush RM Ankrum JA Yang L Stephens SB . Proinflammatory cytokines mediate pancreatic β-cell-specific alterations to golgi integrity via inos-dependent mitochondrial inhibition. Diabetes. (2025) 74:1992–2007. doi: 10.2337/db25-0132 40906541 PMC12585162

[39] Bellin MD . Pancreatogenic diabetes in children with recurrent acute and chronic pancreatitis: Risks, screening, and treatment (mini-review). Front Pediatr. (2022) 10:884668. doi: 10.3389/fped.2022.884668 35558377 PMC9086714

[40] Zhi M Zhu X Lugea A Waldron RT Pandol SJ Li L . Incidence of new onset diabetes mellitus secondary to acute pancreatitis: A systematic review and meta-analysis. Front Physiol. (2019) 10:637. doi: 10.3389/fphys.2019.00637 31231233 PMC6558372

