# Supplementary material for: Combining MRI dual-sequence radiomics and clinical parameters predicts post-hypertriglyceridemic acute pancreatitis diabetes mellitus
Source: Front Endocrinol (Lausanne). 2026 Aug 3;17:1887050. doi: 10.3389/fendo.2026.1887050 (PMC13477960; doi:10.3389/fendo.2026.1887050)
Supplement: Supplementary file 1 [file DataSheet1.pdf]

Supplementary Material

1 Supplementary Figures and Tables

1.1 Supplementary Figures

Figure S1. Manual delineation of regions of interest (ROIs) on dual-sequence MRI.

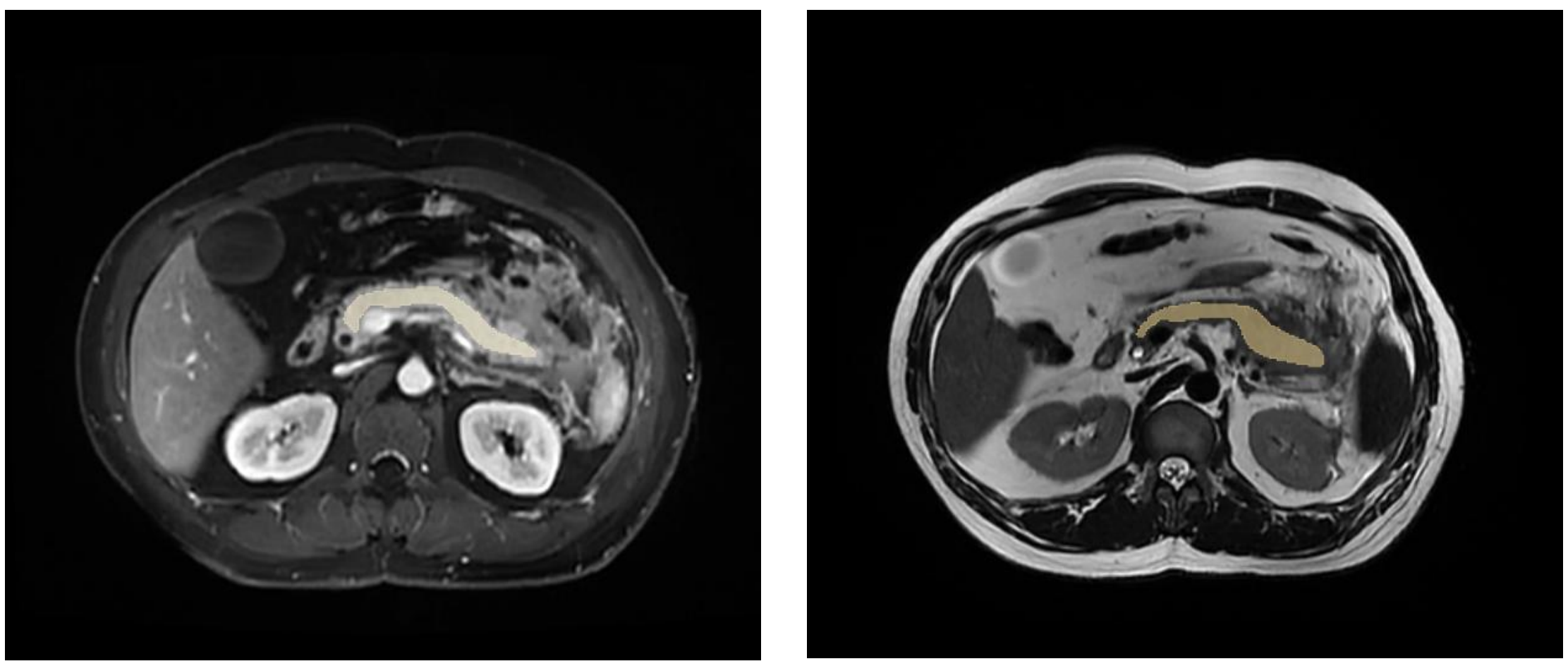

(A) (B)

Representative dual-sequence MR images showing the slice-by-slice manual segmentation of the pancreatic parenchyma. The ROI masks were placed on (A) T2-weighted imaging (T2WI) and (B) late arterial phase contrast-enhanced T1-weighted imaging (CE-T1WI). To minimize contamination, necrotic areas, large peripancreatic vessels, and extraneous tissues were explicitly excluded from the final volumes.

Figure S2. Precision-recall curves of the combined model for predicting HTG-PPDM-A in the internal training and testing cohorts.

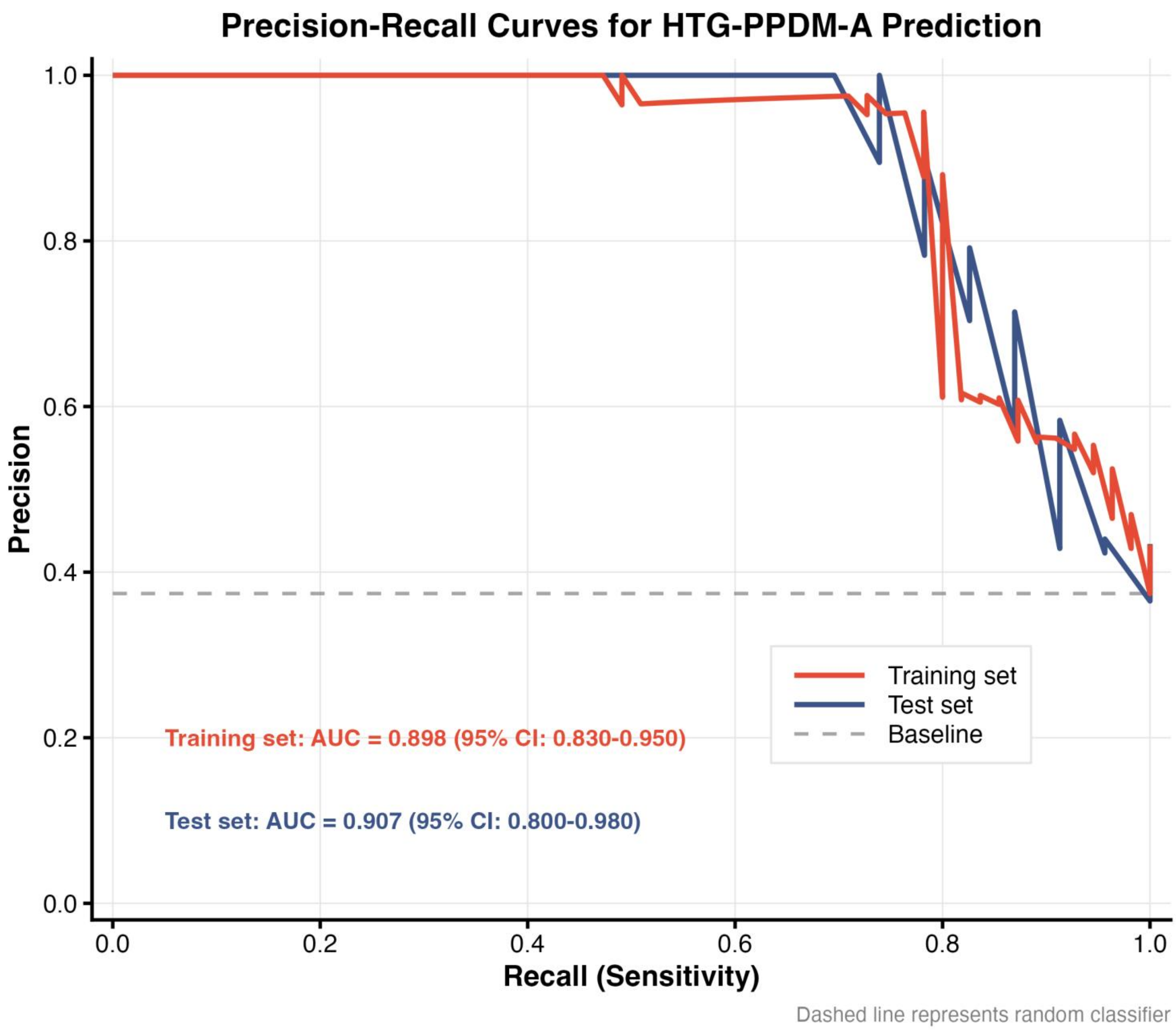

Precision-recall (PR) curves were constructed to evaluate model performance under class imbalance. The PR-AUC values were 0.898 (95% CI: 0.830–0.950) in the internal training cohort and 0.907 (95% CI: 0.800–0.980) in the internal testing cohort. The dashed diagonal line represents the performance of a random classifier (baseline). PR-AUC: area under the precision-recall curve; HTG-PPDM-A: hypertriglyceridemia-associated post-acute pancreatitis diabetes mellitus; CI: confidence interval.

Figure S3. Calibration curves of the combined model in the training, testing, and external validation cohorts.

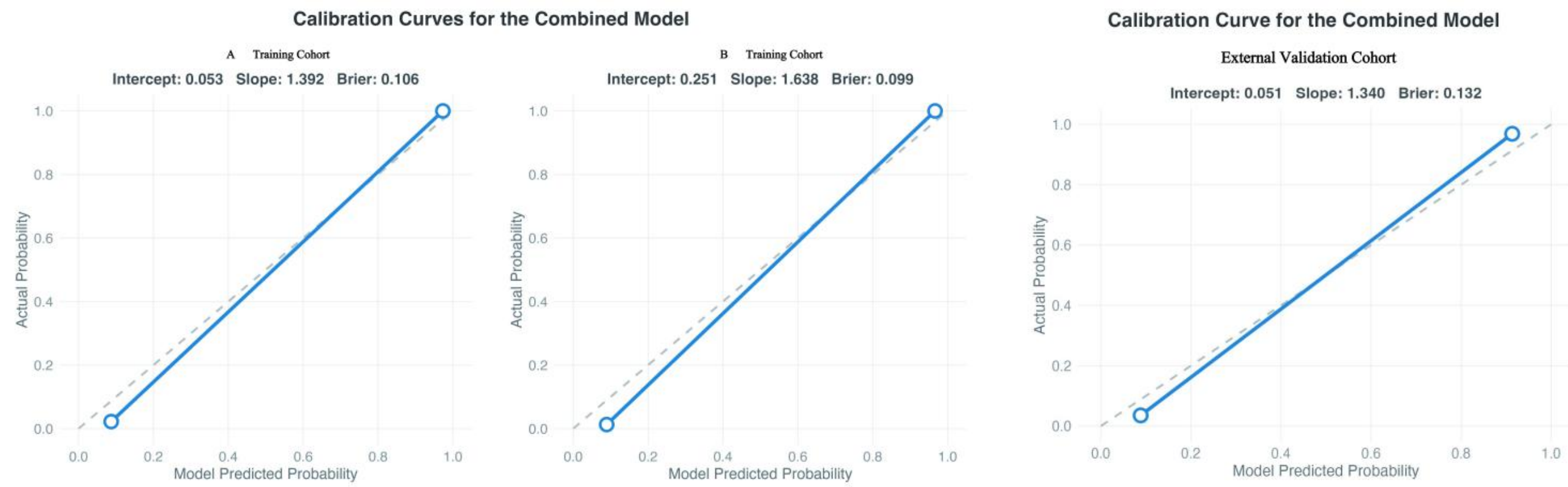

Calibration curves were constructed to evaluate the agreement between predicted probabilities and observed outcomes. The Brier scores were 0.106, 0.099, and 0.132 for the training, testing, and external validation cohorts, respectively, with corresponding intercepts of 0.053, 0.251, and 0.051, and slopes of 1.392, 1.638, and 1.340. The diagonal dashed line represents perfect calibration (ideal prediction). In a well-calibrated model, the calibration curve closely follows the diagonal line. Brier scores < 0.25 are generally considered acceptable for clinical prediction models. Brier: Brier score; HTG-PPDM-A: hypertriglyceridemia-associated post-acute pancreatitis diabetes mellitus.

Figure S4. Ten-fold cross-validation ROC curves for the combined clinical-radiomics model in the internal training cohort.Supplementary .

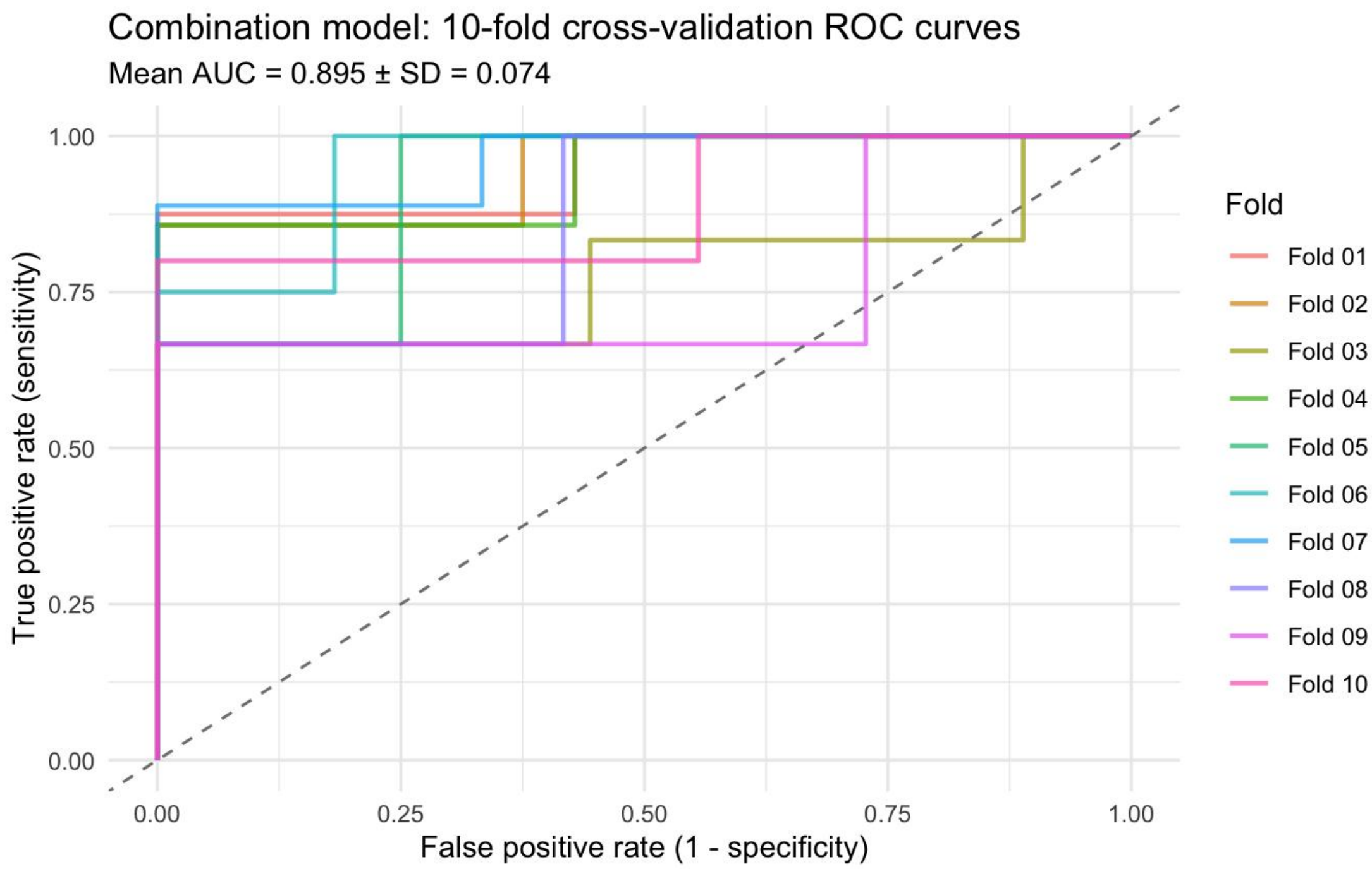

Receiver operating characteristic (ROC) curves from ten-fold cross-validation of the combined model in the internal training cohort. Light gray curves represent individual folds, and the solid blue curve denotes the mean ROC with the shaded area indicating the 95% confidence interval. The model achieved a mean area under the ROC curve (AUC) of 0.895±0.074 (coefficient of variation, 8.30%), with fold-specific AUCs ranging from 0.758 to 0.963. The mean sensitivity was 0.755 and mean specificity was 0.941. The diagonal dashed line indicates no discrimination (AUC = 0.5).

1.2 Supplementary Tables

Table S1 MRI acquisition parameters for the main and verification cohorts.

Parameters	Derivation Cohort	External Validation Cohort
Equipment Manufacturer / Model	United Imaging Healthcare uMR790	GE Healthcare Discovery 750
Magnetic field strength	3.0T	3.0T
Coil Type	16-channel body phased array coil	32-channel body phased array coil
Subject preparation	Fast for ⩾ 4 hours prior to scanning and perform breathing exercises	Fast for ⩾ 4 hours prior to scanning and perform breathing exercises
Posture and Breath Control	Supine position, head-first, using respiratory gating to minimize motion artifacts	Supine position, head-first, using respiratory gating to minimize motion artifacts
Contrast agent	Gadobutrol (GdBOPTA), 0.1 mmol/kg, injection rate 2.5 mL/s	Gadobutrol (GdBOPTA), 0.1 mmol/kg, injection rate 2.5 mL/s
Incorporation of radiomics analysis into the sequence	Late arterial phase (30 seconds)	Late arterial phase (30 seconds)
CE-T1-weighted sequence type	Axial Gradient Echo Sequence	Axial Phase-Shift Gradient Echo Water-Fat Separation T1-Weighted Image
Repeat Time (TR, ms)	3.0	3.8
Echo Time (TE, ms)	1.3	1.7
Field of view (FOV, cm)	40 × 30	36 × 36
Matrix size	157 × 133	224 × 192
Layer thickness / Layer spacing (mm)	1.5 / 0.0	5.2 / 0.0
T2-weighted sequence type	Fast Spin Echo	Fast Spin Echo
Repeat Time (TR, ms)	8000	6000
Echo Time (TE, ms)	116	120
Field of view (FOV, cm)	38 × 30	34 × 34
Matrix size	256 × 171	320 × 256
Layer thickness / Layer spacing (mm)	5.0 / 2.0	6.0 / 1.0
Quality Control	Two senior radiologists performed blinded reading, excluding cases with severe motion artifacts or	Consistent with the main queue

	missing key phase images	

Notes: All examinations were performed on 3.0 T MRI scanners using body phased-array coils with standardized patient preparation (fasting and breathing training).

Image quality was independently evaluated by two senior radiologists; cases with severe motion artifacts or missing critical phases were excluded.

Despite differences in scanner manufacturers and coil channels, consistent imaging planes and contrast phases were maintained between the two cohorts.

Table S2. Hyperparameter configurations of the primary random forest model and the three comparative classifiers.

Classifier	Key Hyperparameters	Decision Threshold
RF	n_estimators = 100, max_depth = 2, min_samples_split = 2, min_samples_leaf = 1, criterion = gini, class_weight = None	0.5
SVM	Kernel = RBF, C = 1.0, γ = 0.01, class_weight = None	0.5
LR	Penalty = L2, C = 1.0, tol = 1×10^{-4} , solver = default	0.5
GP	Kernel = RBF, length_scale = 1.0, n_restarts_optimizer = 10	0.5

Notes:RF, random forest; SVM, support vector machine; LR, logistic regression; GP, Gaussian process. The hyperparameters for RF were chosen based on preliminary experiments; other classifiers were implemented using default settings from the scikit-learn library (version 1.2.2). The decision threshold of 0.5 was used for binary classification in all models.

Table S3. Derivation cohort patient characteristics.

	HTG-AP-PPDMA (n=78)	HTG-AP-nPPDMA (n=132)
**General data**		
Age, years	44.50(37.75,52.00)	43.00(36.00,51.00)
Sex (male/female), n (%)	28(35.90)/50(64.10)	42(31.82)/90(68.18)
Alcohol consumption, n (%)	35(44.87)	57(43.18)
BMI, kg/m²	26.00(23.85,27.33)	26.4.00(24.25,29.75)
Smoking history, n (%)	30(38.46)	52(39.39)
Fatty liver, n (%)	55(70.51)	76(57.58)
Hypertension, n (%)	18(23.08)	30(22.73)
Recurrent attacks, n (%)	24(30.77)	23(17.42)
Biliary stones, n (%)	9(11.54)	20(15.15)
**Disease severity and complications**		
AP Severity	27(34.62)	15(11.36)
Pleural effusion, n (%)	47(60.26)	78(59.09)
Ascites, n (%)	34(43.59)	49(37.12)
Peritonitis, n (%)	38(48.72)	56(42.42)
SIRS, n (%)	31(39.74)	37(28.03)
Length of hospital stay, days	12(9,18)	9(6,11)
**Laboratory parameters**		
Blood glucose (mmol/L)	10.96(7.41,14.43)	10.96(6.66,13.97)
HDL-C(mmol/L)	0.66(0.48,0.89)	0.72(0.60,0.89)
hs-CRP(mg/L)	114.07(68.64,170.59)	69.07(24.63,121.21)
Lipase (U/L)	204.50(76.75,606.5)	379.50(100.25,951.25)
TG (mmol/L)	7.62(4.00,15.50)	9.04(4.71,17.69)
Amylase (U/L)	96.50(44.75,273)	144.00(57.25,419.25)
LDL-C(mmol/L)	2.26(1.17,3.54)	2.225(1.18,3.24)
WBC count ($\times 10^9$ /L)	13.34(10.09,15.95)	12.81(9.38,15.56)
Neutrophil percentage, %	84.10(83.19,85.23)	83.86(83.22,85.91)
Serum calcium (mmol/L)	2.34(2.15,2.44)	2.31(2.19,2.45)
Total cholesterol (mmol/L)	6.36(4.53,7.81)	6.61(4.96,8.73)

Note: a—χ^2 value; b—t value; c—Z value, a Data are expressed as number (%). Group comparisons were performed using the χ^2-test or Fisher's exact test, as appropriate. b Data are expressed as mean ± standard deviation. Group comparisons were performed using the independent samples t test. c Data are expressed as median (interquartile range). Group comparisons were performed using the Mann-Whitney U-test. $P < 0.05$ was considered statistically significant.

Table S4. External validation cohort characteristics.

	HTG-AP-PPDMA (n=42)	HTG-AP-nPPDMA (n=77)	statistical value	P value
**General data**				
Age, years	47.29 ± 11.204	44.67 ± 11.860	1.176[b]	0.242
Sex (male/female), n (%)	14(33.33)/28(66.67)	18(23.08)/60(76.92)	1.469[a]	0.54
Alcohol consumption, n (%)	19(45.24)	32(41.03)	0.198[a]	0.656
BMI, kg/m²	25.766 ± 3.976	26.167 ± 3.185	-0.602[b]	0.548
Smoking history, n (%)	21(50)	33(42.31)	0.653[a]	0.419
Fatty liver, n (%)	29(69.05)	40(51.28)	3.526[a]	0.06
Hypertension, n (%)	13(30.95)	7(8.97)	9.495[a]	0.002
Recurrent attacks, n (%)	15(35.71)	16(20.51)	3.293[a]	0.07
Biliary stones, n (%)	10(23.81)	20(25.61)	0.049[a]	0.825
**Disease severity and complications**				
AP Severity	10(23.81)	4(5.13)	9.245[a]	0.002
Pleural effusion, n (%)	24(57.14)	15(19.23)	17.887[a]	<0.001
Ascites, n (%)	22(52.38)	14(17.95)	15.413[a]	<0.001
Peritonitis, n (%)	18(42.86)	22(28.21)	2.637[a]	0.104
SIRS, n (%)	20(47.62)	23(29.49)	3.903[a]	0.048
Length of hospital stay, days	11(8.00,17.00)	12(8.00,15.00)	- 0.245[c]	0.806
**Laboratory parameters**				
Blood glucose (mmol/L)	8.04(6.77,9.94)	7.2(6.22,8.32)	-2.094[c]	0.036
HDL-C(mmol/L)	0.80(0.68,0.96)	0.76(0.65,1.00)	-0.270[c]	0.787
hs-CRP(mg/L)	42.23(15.15,81.15)	22.60(7.91,97.26)	-1.161[c]	0.246
Lipase (U/L)	252.7.00(138.00,859.65)	373.00(152.25,933.60)	-0.481[c]	0.63
TG (mmol/L)	4.50(2.34,6.57)	4.09(1.71,9.15)	-0.303[c]	0.762
Amylase (U/L)	107.00(63.75,422.25)	185.60(97.75,527.25)	-1.678[c]	0.093
LDL-C(mmol/L)	2.512 ± 0.642	2.264 ± 0.584	2.143[b]	0.034
WBC count ($\times 10^9$ /L)	12.009 ± 3.849	14.11 ± 5.183	-2.409[b]	0.018
Neutrophil percentage, %	84.43(83.87,85.34)	83.64(82.75,83.33)	- 2.839[c]	0.005
Serum calcium (mmol/L)	2.36(2.22,2.48)	2.27(2.11,2.41)	-2.224[b]	0.026
Total cholesterol (mmol/L)	4.66(4.08,6.14)	4.68(4.11,5.74)	-0.311[c]	0.765

Note: a—χ^2 value; b—t value; c—Z value, a Data are expressed as number (%). Group comparisons were performed using the χ^2 -test or Fisher's exact test, as appropriate. b Data are expressed as mean ± standard deviation. Group comparisons were performed using the independent samples t test. c Data are expressed as median (interquartile range). Group comparisons were performed using the Mann-Whitney U-test. P < 0.05 was considered statistically significant.

Table S5. Performance comparison of different classifiers across model types in the training and test cohorts.

Model Type	Classifier	Cohort	AUC (95% CI)	Accuracy	Sensitivity	Specificity
Combined	RF	Training	0.905 (95%CI: 0.847–0.954)	0.902	0.782	0.581
Combined	SVM	Training	0.856 (95%CI: 0.791–0.922)	0.902	0.762	0.527
Combined	LR	Training	0.856 (95%CI: 0.796–0.922)	0.880	0.742	0.509
Combined	GP	Training	0.871 (95%CI: 0.811–0.932)	0.891	0.742	0.491
Combined	RF	Test	0.904 (95%CI: 0.803–0.984)	0.975	0.889	0.739
Combined	SVM	Test	0.617 (95%CI: 0.439–0.796)	0.875	0.714	0.435
Combined	LR	Test	0.712 (95%CI: 0.564–0.860)	0.875	0.698	0.391
Combined	GP	Test	0.685 (95%CI: 0.526-0.843)	0.875	0.698	0.391
Radiomics	RF	Training	0.848 (95%CI: 0.783–0.914)	0.902	0.782	0.581
Radiomics	SVM	Training	0.781 (95%CI: 0.703-0.860)	0.902	0.762	0.527

Radiomics	LR	Training	0.784 (95%CI: 0.707-0.861)	0.880	0.742	0.509
Radiomics	GP	Training	0.785 (95%CI: 0.708-0.863)	0.891	0.742	0.491
Radiomics	RF	Test	0.810 (95%CI: 0.693–0.927)	0.950	0.794	0.522
Radiomics	SVM	Test	0.733 (95%CI: 0.590–0.877)	0.875	0.714	0.435
Radiomics	LR	Test	0.748 (95%CI: 0.609-0.888)	0.875	0.698	0.391
Radiomics	GP	Test	0.766 (95%CI: 0.632-0.900)	0.875	0.698	0.391
Clinical	RF	Training	0.817 (95%CI: 0.734–0.890)	0.924	0.748	0.455
Clinical	SVM	Training	0.765 (95%CI: 0.678–0.852)	0.913	0.755	0.491
Clinical	LR	Training	0.776 (95%CI: 0.693–0.859)	0.891	0.769	0.564
Clinical	GP	Training	0.771 (95%CI: 0.688–0.854)	0.924	0.755	0.473
Clinical	RF	Test	0.751 (95%CI: 0.612–0.889)	0.950	0.746	0.391
Clinical	SVM	Test	0.617	0.980	0.825	0.522

			(95%CI: 0.439–0.796)			
Clinical	LR	Test	0.712 (95%CI: 0.564–0.860)	0.950	0.794	0.521
Clinical	GP	Test	0.685 (95%CI: 0.526-0.843)	0.981	0.825	0.522

Notes: AUC, area under the receiver operating characteristic curve; CI, confidence interval; RF, random forest; SVM, support vector machine; LR, logistic regression; GP, Gaussian process. For each model type (clinical, radiomics, and combined), the best-performing classifier in each cohort is highlighted in bold. All performance metrics are presented as point estimates with 95% confidence intervals in parentheses.
